# NLP modeling recommendations for restricted data availability in clinical settings

**DOI:** 10.1186/s12911-025-02948-2

**Published:** 2025-03-07

**Authors:** Fabián Villena, Felipe Bravo-Marquez, Jocelyn Dunstan

**Affiliations:** 1 Department of Computer Science, Universidad de Chile, Santiago, Chile; 2 Millennium Institute Foundational Research on Data, Santiago, Chile; 3 Facultad de Odontología, Universidad San Sebastián, Santiago, Chile; 4 National Center for Artificial Intelligence, Santiago, Chile; 5 Department of Computer Science, Pontificia Universidad Católica de Chile, Santiago, Chile; 6 Institute for Mathematical and Computing Engineering, Pontificia Universidad Católica de Chile, Santiago, Chile

**Keywords:** Artificial intelligence, Natural language processing, Data availability

## Abstract

**Background:**

Clinical decision-making in healthcare often relies on unstructured text data, which can be challenging to analyze using traditional methods. Natural Language Processing (NLP) has emerged as a promising solution, but its application in clinical settings is hindered by restricted data availability and the need for domain-specific knowledge.

**Methods:**

We conducted an experimental analysis to evaluate the performance of various NLP modeling paradigms on multiple clinical NLP tasks in Spanish. These tasks included referral prioritization and referral specialty classification. We simulated three clinical settings with varying levels of data availability and evaluated the performance of four foundation models.

**Results:**

Clinical-specific pre-trained language models (PLMs) achieved the highest performance across tasks. For referral prioritization, Clinical PLMs attained an 88.85 % macro F1 score when fine-tuned. In referral specialty classification, the same models achieved a 53.79 % macro F1 score, surpassing domain-agnostic models. Continuing pre-training with environment-specific data improved model performance, but the gains were marginal compared to the computational resources required. Few-shot learning with large language models (LLMs) demonstrated lower performance but showed potential in data-scarce scenarios.

**Conclusions:**

Our study provides evidence-based recommendations for clinical NLP practitioners on selecting modeling paradigms based on data availability. We highlight the importance of considering data availability, task complexity, and institutional maturity when designing and training clinical NLP models. Our findings can inform the development of effective clinical NLP solutions in real-world settings.

**Supplementary Information:**

The online version contains supplementary material available at 10.1186/s12911-025-02948-2.

## Introduction

Clinical narratives provide a unique window into a patient’s medical history and progression, and to use these health-related documents in clinical decision support systems, it is necessary to machine-understand text. The area of artificial intelligence devoted to the interaction between humans and machines through language is called Natural Language Processing (NLP) [1]. Classical tasks in clinical NLP are text classification, entity recognition, summarization, and question answering, to mention some [2]. However, working in a specific domain such as medicine poses unique challenges due to its particular jargon, annotation requirements, source variability, and limited data availability [3]. In fact, access to clinical text data is typically highly restricted due to consent requirements, patient privacy concerns, secure access, and sanitization processes [4]. Computational techniques like cryptography and anonymization can help preserve secure access and privacy. Legal processes, such as ethical committees, are also necessary to ensure lawful informed consent.

In addition to the inherent challenges of clinical NLP, disparities in language representation further exacerbate data scarcity issues for underrepresented languages. Many low-resource languages are underrepresented in pre-trained multilingual models, which limits the development of robust NLP tools [5]. Similar challenges affect the English language. In the UK, barriers such as the limited availability of labeled datasets and legal restrictions on data sharing hinder clinical NLP progress [6]. Likewise, German clinical NLP faces obstacles due to the scarcity of publicly available datasets, privacy concerns, and the difficulties associated with working with de-identified data [7]. These challenges underline the global need for public datasets, data-sharing protocols, and domain adaptation strategies to overcome data scarcity and advance clinical NLP across languages and domains.

In terms of NLP techniques, the paradigm used to be the use of recurrent neural networks (RNNs), which preserved the sequence nature of language in the representation of meaning [8, 9]. One drawback of RNNs is their limited parallelizability, resulting in prolonged training times. Furthermore, as the sequence lengths grow, there is a tendency for information gathered at distant time steps to vanish due to inherent memory limitations. Nowadays, the emergence of the Transformer architecture completely ditches the recurrence of the architecture but also preserves word order by learning dependences without regard to their distance in the sentences [10]. With its attention mechanism, this architecture reaches state-of-the-art in multiple NLP tasks such as text classification [11], sentiment analysis [11], dependency parsing [12], machine translation [13], and named entity recognition [14]. In the following paragraphs, we introduce the fundamental concepts needed to understand the experiments presented in this paper.

### Pretrained language models (PLMs)

PLMs are language models (LMs) that were trained using self-supervised techniques over large *corpora* of unannotated text to transfer learning from the knowledge gathered in the pre-training to downstream task-specific models [15]. Early methods for PLMs consisted of static word embeddings, which were distributed word representations learned using algorithms such as Word2Vec [16] or GloVe [17], and these embeddings were standard initialization parameters for deep learning architectures to solve NLP tasks. There has been a shift towards dynamic or context-aware word embeddings, which solves the problem of static word embeddings that do not consider word polysemy. These context-aware word embeddings were initially composed using RNNs [18] such as in ELMo [19], but currently, they are based on the Transformer architecture and use web-scale unannotated text to be trained. The *de facto* standard for pre-trained Transformer-based context-aware models is BERT [20] and BERT-alike models such as RoBERTa [21] and DeBERTa [21]. This language model learns bidirectional contexts, conditioning on both left and right contexts in deep stacked layers. Using BERT as a base architecture, domain-specific models have arisen, such as PubMedBERT [22], a PLM for the biomedical domain in English, and Spanish biomedical and clinical RoBERTa [23], a RoBERTa-based PLM for the clinical domain in Spanish.

### Large language models (LLMs)

LLMs are PLMs with a significantly larger model size scale [24]. For example, the PLM BERT has $0.3 \times 10^{9}$ parameters while the LLM GPT-3 has $175 \times 10^{9}$ parameters [25]. It has been found that scaling PLMs improves the performance of the models on downstream tasks [26]. Although this is true, other surprising and more important behaviors in solving complex tasks appear at LLM scales, called *emergent abilities*. Emergent abilities are aptitudes not present in small models but arise in LLMs [27] and include *in-context learning*, where a model can generate expected outputs to natural language instructions without additional training, *instruction following*, where a model fine-tuned using natural language instructions performs well on unseen tasks that are also described in the form of instructions and *step-by-step reasoning*, where a model can solve complex problems by instructing the model involving intermediate reasoning steps for deriving the final answer. GPT-3, a closed-source privative LLM, formally introduced the concept of in-context learning, and from there, subsequent models have appeared, such as open-source models Galactica [28], a $120 \times 10^{9}$ parameters model and LLaMA 3.1 [29], a $405 \times 10^{9}$ parameters model. It is worth noting that ChatGPT, a significant milestone among LLMs, differs from general pretrained or foundation models such as GPT-3 by virtue of its instruction-tuned architecture, refined through Reinforcement Learning with Human Feedback (RLHF) [30]. Although ChatGPT remains a closed-source, proprietary assistant-style LLM, its superior conversational abilities have led to widespread use among the general public, illustrating how instruction tuning can substantially enhance usability and performance in real-world scenarios.

### Fine-tune and predict paradigm of PLMs

The primary adaptation method for adjusting PLM to downstream tasks is fine-tuning, where a task-specific layer is concatenated to the output of the PLM [31]. This method was proposed in the Universal Language Model Fine-Tuning (ULMFiT) framework as a transfer learning technique for domain-specific NLP, achieving state-of-the-art performances in multiple NLP tasks [32]. Even though the fine-tuning paradigm has been well described for adapting PLMs, LLMs have significantly higher computational complexity due to their unprecedented scale. For this reason, some special techniques have been developed, such as Parameter-Efficient Fine-Tuning (PEFT), where a small set of parameters are trained to enable a model to perform the new task [33], showing improvements over in-context learning [34] .

### Pre-train, prompt and predict paradigm of LLMs

The principal approach for interfacing with LLMs is through prompting, which are instructions in natural language issued to LLMs to adapt them to new scenarios with few or no labeled data [24] by exploiting the emergent ability of in-context learning. This new NLP paradigm created a new field of prompt engineering, where prompting templates are created to achieve the most effective performance on downstream tasks [35]. There is mixed evidence comparing fine-tuning vs. in-context learning, whereas in some tasks such as in biomedical information extraction [36] or out-of-domain generalization [37], fine-tuning outperforms in-context learning; in other tasks, such as code intelligence [38], in-context learning outperforms fine-tuning.

### Domain adaptation

It is well-established that using closer-to-the-domain LMs for fine-tuning downstream tasks significantly improves model performance [22, 23, 39]. One of the most widely used paradigms in NLP is the fine-tuning of PLMs, though this framework offers multiple approaches for optimization. The first approach involves using an existing PLM and either directly fine-tuning it for the downstream task or further pre-training it on domain-specific unlabeled data before fine-tuning. Alternatively, a second approach involves pretraining a language model entirely from scratch using domain-specific unlabeled data, followed by fine-tuning it with task-labeled data, mirroring the process of the first approach. While the second approach may be useful when no pretrained models exist, it is far less common due to the substantial data and computational requirements for both pre-training and fine-tuning.

Some paradigms described above require at least some task-labeled data, but there are some settings where no data is available; for these cases, the prompt and predict paradigm is valid, where an instruction-tuned causal LLM is prompted in natural language to act as an NLP-based model with few or zero examples given [35], exploiting its in-context learning ability. This framework is also an option to consider when building NLP-based models.

**Fig. 1:**
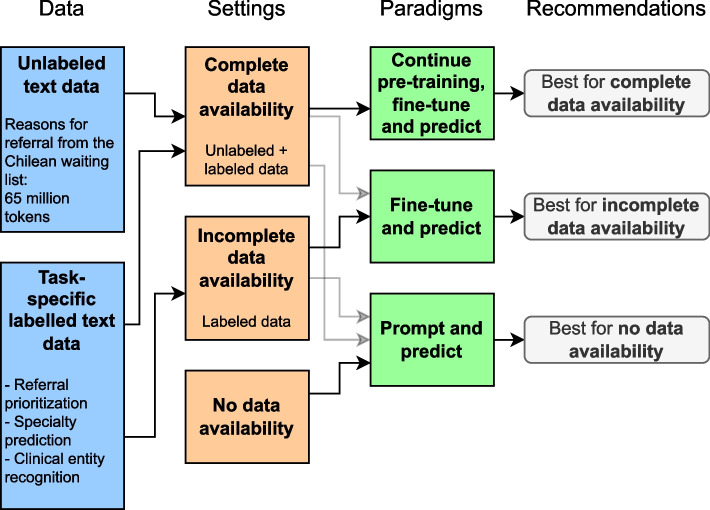
An overview of the compatibilities between available data, settings, NLP paradigms, and recommendations derived from this study. The figure illustrates the flow from data availability (blue boxes) to settings (orange boxes), paradigms (green boxes), and corresponding recommendations (gray boxes). Solid arrows indicate the recommended path for each scenario, while dashed arrows represent alternate tested approaches. For example, the “Continue pre-training, fine-tune and predict” paradigm is best suited for complete data availability, while “Prompt and predict” is recommended in scenarios with no data availability

Data access can be limited in clinical environments due to privacy concerns or interoperability issues, resulting in varying data availability settings. On one hand, some settings offer abundant task-labeled and unannotated data, enabling the application of all NLP modeling paradigms. On the other end, data access may be incomplete or entirely restricted, forcing the use of specific paradigms tailored to these constraints. Figure 1 provides a comprehensive overview of these data availability settings, their compatibility with the described NLP modeling paradigms, and recommendations we validated in this paper.

### Problem

A situation arises when there is an asymmetry in data availability, or no data is available. In some cases, there is only task-labeled data, only domain-specific unlabeled data, or no data is available at all. Even though multiple paradigms exist for NLP modeling in clinical environments, the compatibility between data availability and the NLP modeling paradigm regarding gains in performance still needs to be explored.

### Solution

We conducted an experimental analysis to evaluate the performance of addressing clinical NLP tasks in Spanish using various combinations of data availability and NLP modeling paradigms. We also formulated empirical recommendations for clinical NLP modeling based on data availability.

## Methods

We intentionally limited data access to evaluate its impact on the performance of multiple clinical NLP modeling paradigms and foundation models. Each restricted setting was based on a real-world simulated clinical environment.

### Simulated settings

To mimic clinical settings regarding data availability, we simulated multiple settings with varying levels of data availability. We divided the data into two categories: task-specific labeled data, which can be used to fine-tune models and environment-specific unlabeled data, which can be used to continue the pre-training of the foundation models. The overall environment we are located in is a Chilean public health institution analyzing waiting list data, where the explanation of why the patient is waiting is in the form of free text, and from that dataset, multiple tasks need to be solved [40]. Multiple reasons can restrict data availability; for example, data availability for model training can be restricted due to legal and privacy reasons or because the task trying to be solved still does not have sufficient examples due to its recent appearance.

#### Unannotated data

The unlabeled data we used to continue the pre-training of the foundation models was the complete set of reasons for referral contained in the Chilean waiting list and is comprised of 3,365,476 documents, totalling 65,891,568 tokens with a vocabulary size of 513,315 types.

#### Complete data availability

In this data availability setting, unlabeled unstructured free-text data to continue the pre-training and task-specific labeled data are also available to fine-tune foundation models. This setting can be seen at a large healthcare provider or at a country-level public health institution such as a ministry of health, where data policies are well established, and patients must consent that their data can be used to tune machine learning models.

#### Incomplete data availability

In this data availability setting, only task-specific labeled data is available to fine-tune foundation models. The lack of unlabeled unstructured free-text data to continue the pre-training may be attributed to the fact that the provided is only acquiring data for the specific task and does not have access to close-to-the-environment unlabeled text data or according to data policies, the provider cannot merge patient data from a different source, other than the source of the task data. This setting can be seen at a medium-sized healthcare provider where the data warehousing methods are not implemented or the provider only has access to specific and segmented data sources due to the lack of interoperability.

#### No data availability

In this data availability setting, there is no unlabeled unstructured free-text data to continue the pre-training nor task-specific labeled data to fine-tune foundation models. The absence of data can be attributed to the lack of access to the electronic health record (EHR) database or policies that forbid patient data usage to tune machine learning models. This setting can be seen in a healthcare provider using an external EHR service that forbids access to the underlying database, or the provider wants to solve a new task where data is not yet available.

### Clinical NLP tasks

To measure the impact of data availability on the performance of clinical NLP modeling, we used multiple clinical NLP tasks, where each is under the same environment of the analysis of unstructured waiting list data.

#### Referral prioritization

Different methods exist to prioritize patient selection to process the waiting list more fairly, and we modeled the patient prioritization through the classification of each referral regarding its state according to the Chilean Explicit Health Guarantees law (GES in Spanish), which states that specific health problems must be guaranteed to be resolved within a particular time frame. This task requires a binary classification modeling technique. The dataset [41] contains 1,701,582 examples in the training subset, 485,649 in the test subset and 242,746 in the validation subset. The baseline performance of this task reported by [41] is a macro F1 score of 80 %. The dataset is available at https://huggingface.co/datasets/fvillena/ges.

#### Referral speciality classification

Each referral contained on the waiting list corresponds to a specific medical speciality. This task involves the prediction of the corresponding medical speciality given the free-text description of the reason for referral contained on the waiting list record. This task requires a multilabel modeling technique with a label space size of 48 classes. The dataset contains 3,401,173 examples in the training subset, 971,764 in the test subset and 485,882 in the validation subset. The dataset is available at https://huggingface.co/datasets/fvillena/spanish_diagnostics.

#### Named entity recognition

Clinical named entity recognition is a subtype of named entity recognition in which entities of clinical interest are extracted from unstructured free-text sources. This dataset [40, 42] is annotated with eleven different clinical entity classes and was modeled as a token classification problem, where each of the tokens of the referrals is classified into one of the eleven clinical entity classes. The dataset contains 7987 documents in the training subset, 987 in the test subset and 887 in the validation subset. The baseline performance of this task reported by [42] is a micro F1 score of 80 %. The dataset is available at https://huggingface.co/datasets/plncmm/wl.

### Foundation models

We used multiple foundation models as a basis to solve the clinical NLP tasks. The attributes used to select the foundation models were the language, domain and modeling technique.

We selected a diverse set of models reflecting increasing levels of domain adaptation: starting with a multilingual model not specifically trained for Spanish, progressing to a model exclusively trained on Spanish clinical text. For the prompt-and-predict paradigm, we chose a widely used open-weight model to prioritize accessibility and ensure ease of reproducibility in our experiments.

#### XLM-RoBERTa

A multilingual version of XLM-RoBERTa masked language model, pre-trained using a self-supervised technique on a *corpus* of 2.5 TB of filtered CommonCrawl raw text data containing one hundred languages [43]. This model is the broadest of all of our selected foundation LMs. This model should be viewed as a baseline where no model is available for the language or the domain. We used the base version of $125 \times 10^{6}$ parameters.

#### Spanish RoBERTa

A Spanish language version of RoBERTa masked language model, pre-trained on a *corpus* of 570 GB of clean and deduplicated text, compiled from the web crawlings performed by the National Library of Spain (Biblioteca Nacional de España) from 2009 to 2019 [44]. This model is only compatible with the language in which the clinical NLP tasks are and is a type of model (regarding language) that should be used when no domain-specific model is available. We used the base version of $125 \times 10^{6}$ parameters.

#### Spanish biomedical and clinical RoBERTa

A Spanish language biomedical and clinical version of RoBERTa masked language model, pre-trained on a *corpus* of several biomedical *corpora* in Spanish, collected from publicly available *corpora* and crawlers, and a real-world clinical *corpus*. The entire *corpus* was comprised of more than 1B tokens, the largest clinical corpus in Spanish [23]. This model is the closest to the domain model we used to solve the tasks, compatible with both language and domain; this should be the best-suited model to solve a domain-specific task. We used the base version of $125 \times 10^{6}$ parameters.

#### Llama

Llama is a causal auto-regressive language model that uses an optimized transformer architecture trained on a *corpus* of publicly available online data comprised of two trillion tokens [45, 46]. This model is the largest we tested but is not domain-adapted in any way, and this is the model we used for in-context learning prediction. Although proprietary LLMs may achieve slightly better performance, the differences are not substantial. Therefore, we chose to use open-weight models to prioritize reproducibility and ensure that our experiments can be easily replicated and extended by the research community. We used the Llama 2 & 3 full-precision Instruct version of $70 \times 10^{9}$ parameters.

### Modeling paradigms

We utilized various NLP modeling paradigms to tackle each clinical NLP task, experimenting with multiple paradigms for some foundational models based on their compatibility. Also, we note the compatibility of each paradigm with each data availability setting.

#### Continue pre-training, fine-tune and predict

This modeling paradigm is the most data-intensive, where we start with an already pre-trained LM checkpoint and continue the pre-training for five epochs with the closer-to-the-environment unannotated data described in “Simulated settings”. Then, with the now environment-adapted LM, we perform a fine-tuning for five epochs to solve each clinical NLP task. We continued the pre-training of all the masked LMs (XLM-RoBERTa, Spanish RoBERTa and Spanish biomedical and clinical RoBERTa) with no modification to the original vocabulary and using model-default hyperparameters using HugginFace’s transformers library [47]. This paradigm is compatible only with the Complete data availability setting. While it might seem that having access to task-labeled data (as in the Incomplete data availability setting) implicitly provides access to textual data for continued pretraining, the dataset size in such scenarios is often too small to yield significant performance improvements through pretraining.

#### Fine-tune and predict

In this paradigm, we started with each of the off-the-shelf masked foundation models (XLM-RoBERTa, Spanish RoBERTa and Spanish biomedical and clinical RoBERTa) and performed fine-tuning for each of the clinical NLP tasks. We used HuggingFace’s AutoModelForSequenceClassification class for the text-classification tasks, and for the NER task, we used the AutoModelForTokenClassification class. We fine-tuned each task using the default model hyperparameters and trained for five epochs using HugginFace’s transformers library [47]. This paradigm is compatible with both complete and incomplete data availability settings.

#### Prompt and predict

In this paradigm, we exploited LLMs’ in-context learning emergent ability through zero-shot and few-shot techniques. We prompted the LLMs Llama 2 and 3 to solve each task and parsed its answer accordingly. For the few-shot technique, we randomly sampled five examples of the training subset of each clinical NLP task. This task is compatible with complete, incomplete and no data availability settings. The prompt templates used to solve each task are available in the Appendix.

### Increasing training data size and its impact on model performance

To better understand the direct impact of the number of training examples, we performed a test in which we truncated the training subset in increasing steps and measured the performance of the fine-tuned model on the complete test subset. We applied this experiment to all settings and masked LMs.

## Results

**Fig. 2:**
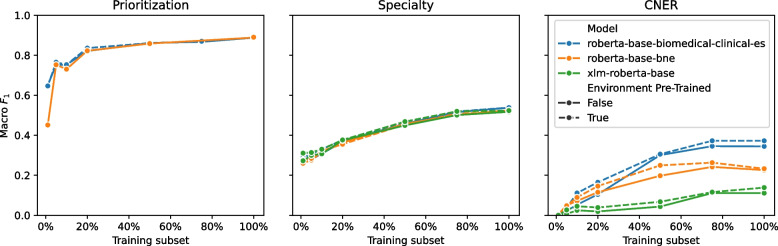
Performance of the models in the downstream tasks by increasing the training data size

**Fig. 3:**
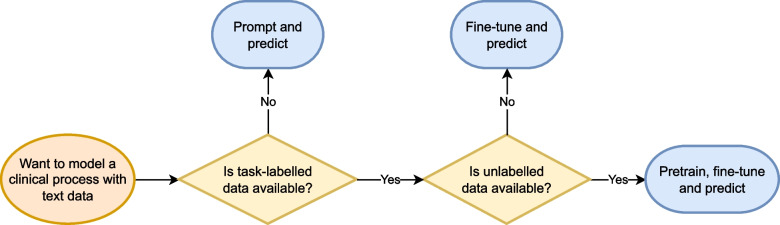
Algorithm for selecting the best modeling paradigm based on data availability

The results for each modeling paradigm solving each clinical NLP task are presented in Table 1. For a more detailed analysis, an extended table presenting additional performance metrics is provided in the Appendix. Additionally, Table 2 presents the training times for each modeling paradigm to offer insights into the computational costs associated with the approaches. The model performances by increasing training data size are presented in Fig. 2 and our modeling recommendations are available at the end of this section and an algorithm for selecting the best paradigm is in Fig. 3.

**Table 1:** Results (macro $F_1$ score^a^ ) for each clinical NLP task and each modeling paradigm

Model & paradigm	Prioritization	Specialty	NER
**xlm-roberta**			
Fine-tune & predict	88.85 %	51.71 %	11.09 %
Cont. pre-train., fine-tune & pred.	89.03 % (+0.18)	52.36 % (+0.65)	13.85 % (+2.76)
**roberta-bne**			
Fine-tune & predict	88.58 %	52.50 %	22.59 %
Cont. pre-train., fine-tune & pred.	88.80 % (+0.22)	51.65 % (−0.85)	23.29 % (+0.70)
**roberta-biomedical-clinical**			
Fine-tune & predict	88.80 %	53.79 %	34.46 %
Cont. pre-train., fine-tune & pred.	88.85 % (+0.05)	53.85 % (+0.06)	37.25 % (+2.79)
**Llama 2**			
Prompt & predict (Zero-shot)	6.49 %	31.41 %	5.31 %
Prompt & predict (Few-shot)	56.70 % (+50.21)	31.91 % (+0.50)	15.44 % (+10.13)
**Llama 3**			
Prompt & predict (Zero-shot)	36.87 %	38.49 %	17.59 %
Prompt & predict (Few-shot)	47.64 % (+10.77)	48.50 % (+10.01)	23.14 % (+5.55)

^a^Macro $F_1$ score is the unweighted average of the $F_1$ scores calculated for each class, treating all classes equally regardless of their frequency

**Table 2:** Training times (in hours) for each clinical NLP task and modeling paradigm. Values in parentheses indicate how many times longer the “Continue pre-training, fine-tune & predict” paradigm takes compared to the “Fine-tune & predict” paradigm. All training times were measured using a single NVIDIA RTX 4090 GPU. It is important to note that the reported times for the “Continue pre-training, fine-tune & predict” paradigm include the continuation of pre-training, which needs to be performed only once and can then be reused for multiple tasks

Model & paradigm	Prioritization	Specialty	NER
**xlm-roberta**			
Fine-tune & predict	3.81	9.39	0.09
Cont. pre-train., fine-tune & pred.	13.56 (3.6x)	19.14 (2.0x)	9.84 (109.3x)
**roberta-bne**			
Fine-tune & predict	4.13	9.46	0.11
Cont. pre-train., fine-tune & pred.	13.88 (3.4x)	19.21 (2.0x)	9.86 (89.6x)
**roberta-biomedical-clinical**			
Fine-tune & predict	4.02	9.68	0.10
Cont. pre-train., fine-tune & pred.	13.77 (3.4x)	19.43 (2.0x)	9.85 (98.5x)

### Clinical PLMs are the best baselines

We tested three encoder models and fine-tuned them to solve each clinical task. Specifically, we tested a multilingual PLM that included Spanish texts in its training data [43], a Spanish-specific PLM trained primarily on non-clinical texts [44], and a specialized PLM trained exclusively on clinical texts in Spanish [23]. Additionally, we used prompting with a multilingual large language model (LLM) to tackle the tasks. Our results showed that the PLM trained solely on clinical texts in Spanish outperformed the others, achieving top scores in two out of three clinical tasks, as shown in the row roberta-biomedical-clinical of Table 1.

### Continual pre-training on local clinical data improves performance

We further pre-trained all three PLMs on our unlabeled clinical text dataset, which serves as a common foundation for all three tasks. This approach simulates a real-world scenario where a healthcare provider needs to tackle multiple tasks while having access to a large corpus of unlabeled text data from various sources. By leveraging this auxiliary data, we observed improvements in the performance of all three models across the three clinical tasks, demonstrating the effectiveness of unsupervised pre-training for enhancing model adaptability and task-specific performance, as shown on the values inside parentheses on the three first rows of Table 1. Continuing the pre-training process added an extra 9.75 hours of GPU time to each model’s fine-tuning, as detailed in Table 2.

### Prompt and predict paradigm is not ready for solving clinical tasks, even with few-shot learning

In addition to fine-tuning a PLM on training data for each clinical task, we explored the prompt and predict paradigm as an alternative approach. This involved providing natural language instructions to an LLM and prompting it to generate answers for each task. Although this paradigm offers a promising framework for tackling complex tasks with little to no data, our results showed that its performance fell short of achieving top results in any clinical tasks we evaluated. We also used few-shot which improves the performance. These results are shown in the two last rows of Table 1.

### The more training data you have, the better

To investigate the impact of training data quantity on fine-tuned model performance, we conducted an experiment where we trained multiple task-specific models using incremental subsets of training data ranging from small to large dataset proportions. Our analysis revealed a positive correlation between the proportion of training data used and the resulting model performance, indicating that increasing the amount of training data leads to improved performance, as shown in Fig. 2.

### Our recommendations

Based on the insights gained from our comprehensive experiments, we distill a set of evidence-based recommendations for clinical NLP practitioners tailored to address the varying levels of data availability commonly encountered in real-world clinical settings. These recommendations aim to guide practitioners on effectively leveraging clinical NLP technologies in diverse environments, from those with abundant data resources to those with limited or no labeled data. We also propose a simple algorithm for selecting the best paradigm, shown in Fig. 3.

Model selection When selecting foundation models, prioritize those that align closely with the target domain. Our results emphasize the significance of domain specificity in achieving optimal performance.
Data utilization In settings with ample access to task-specific labeled data and unlabeled domain-specific text, the pre-train, fine-tune and predict paradigm should be considered. However, given the resource-intensive nature of this approach, practitioners may opt for the fine-tune and predict paradigm, especially when computational resources are constrained.
If no data is available Only in scenarios with no access to labeled data, the prompt and predict paradigm, particularly with few-shot learning, emerges as a practical and effective solution. This approach allows models to leverage general knowledge and adapt to new tasks with minimal labeled examples.
Consideration of task complexity Recognize the inherent complexity of the clinical NLP task at hand. Tasks with lower complexity may achieve near-optimal performance even with minimal access to training data, highlighting the importance of task-specific considerations.
Continuous investigation Clinical NLP is dynamic, and advancements in pre-trained foundation LMs and novel paradigms are frequent. Continuously exploring emerging techniques and adapting to the evolving landscape is essential for staying at the forefront of effective healthcare information extraction.

To support the recommendations, real-world examples demonstrate how each modeling paradigm can be effectively applied in clinical NLP. When large-scale domain-specific text is available, continuing pretraining before fine-tuning enhances model adaptability and improves performance on specialized tasks, as shown in prior work on named entity recognition in Spanish clinical referrals [48]. In scenarios where only task-specific labeled data is present, fine-tuning pre-trained models remains an effective and computationally efficient strategy for improving accuracy in tasks such as named entity recognition and classification, as demonstrated in studies on privacy-preserving occupational health corpora [49]. Finally, in low-resource settings where access to labeled data is limited or unavailable, prompt-based approaches provide a viable alternative by leveraging pretrained language models to extract relevant clinical information with minimal supervision, as seen in recent evaluations of few-shot learning for clinical information extraction [50]. These examples illustrate how aligning modeling choices with data availability constraints can lead to more effective clinical NLP implementations.

## Discussion

Our experiments confirm that clinical-specific PLMs achieve the highest performance on the clinical NLP tasks we evaluated, aligning with previous studies in English [51], Spanish [44], German [52], and Portuguese [53], which demonstrate the superiority of domain-specific PLMs over general-purpose models. Additional multilingual studies reinforce this, such as Gaschi et al. [54], who showed cross-lingual transfer and translation approaches can achieve strong NER results in French and German using multilingual and domain-specific models. Together, these studies underscore the importance of domain-specific adaptation for advancing clinical NLP in diverse languages, supporting our findings.

While LLMs have made progress in the clinical domain [55], there is still a notable gap in the availability of clinical-specific LLMs for languages other than English. Considering data availability, clinical-specific PLMs can be employed with pre-train, fine-tune, and predict and fine-tune and predict paradigms. However, we recommend using the latter, as it balances computational intensity and performance, whereas the former requires substantially more computational resources without yielding significant performance improvements. Furthermore, the pre-train, fine-tune and predict paradigm is less practical for real-world applications due to its high resource demands, making it a more viable option for most clinical NLP use cases.

Our experiments revealed that the pre-train, fine-tune, and predict paradigm yields the best performance when tackling clinical NLP tasks, as other authors have previously suggested [56, 57]. As expected, we found that pre-training a model on unlabeled free-text data from the local environment, starting from a clinical-specific checkpoint and then fine-tuning to solve the tasks, consistently achieved superior performance. Continuing pre-training with unlabeled close-to-the-domain data consistently enhanced model performance, suggesting a reliable approach for incremental improvement. However, despite its superior performance, engineers should be aware that this paradigm comes at a substantial computational cost, requiring significant energy consumption and computational resources, as shown in Table 2. For instance, the continuation of pre-training increased training times for each model by multiple times compared to the fine-tune and predict paradigm alone. These extended training times highlight the need to carefully evaluate the trade-offs between performance gains and computational demands, particularly in environments with limited resources or energy constraints. As such, engineers must carefully weigh the benefits of this paradigm against its costs, particularly in environments where computational resources are limited or energy efficiency is a concern. Ultimately, this approach may be most suitable for applications where high-stakes decision-making necessitates optimal performance but may not be the most practical choice for resource-constrained environments or cases where performance improvements are not critical.

Despite the notable success of LLMs in medical benchmarks [58], we were surprised to find that their performance on our clinical tasks was suboptimal, particularly in few-shot settings. While few-shot learning offers potential in data-scarce scenarios, as highlighted by Ge et al. [59], progress in biomedical NLP has been limited, with few-shot methods consistently underperforming relative to other approaches. Similarly, Moradi et al. [60] found that GPT-3, despite its near state-of-the-art performance in open-domain few-shot tasks, performed poorly in biomedical NLP compared to fine-tuned models, which benefits from domain-specific pretraining. We found that LLMs in few-shot scenarios were unable to compete with fine-tuned PLMs for our clinical tasks. This disparity further reinforces the need for advancements in few-shot learning methods that better align with the complexities and data limitations of clinical domain.

To improve performance with the prompt and predict paradigm, one approach could be utilizing clinical-specific LLMs, such as MEDITRON [55] or BioMistral [61]. However, these models are currently only available for the English language. Alternatively, fine-tuning the model on local data using adapters like LoRA, which have shown promise in English-language clinical applications [62], may offer a viable solution.

Another promising strategy is leveraging agentic systems, which integrate one or more LLMs with access to external tools such as a programming language interpreter or web search to enhance task performance [63]. These LLM-based agents can function as intelligent entities, interacting dynamically with other agents or tools, processing complex workflows, and addressing challenges in specific clinical contexts. For example, Li et al.[64] proposed a multi-agent system that mimics real-world ICD coding workflows, integrating distinct roles (e.g., physician, coder, reviewer) and leveraging LLMs to outperform traditional prompting techniques in both common and rare code accuracy.

We empirically validated the positive correlation between training data size and model performance, confirming that increasing the amount of training data can lead to better performance, as shown in Fig. 2. Notably, our results showed that the models’ performance did not continue to improve indefinitely but instead reached a saturation point even before all available training data was utilized. This phenomenon was most pronounced in the prioritization clinical NLP task, where even a minimal amount of training data was sufficient to achieve near-optimal performance, suggesting that this task has relatively low complexity. In contrast, the Specialty prediction task exhibited a more complex relationship between training data availability and performance, with a nearly linear correlation indicating that access to sufficient training data is critical for achieving high accuracy on this task. Our findings highlight the importance of considering the specific data requirements for each task when designing and training clinical NLP models.

To effectively implement the clinical NLP paradigms presented in this work, healthcare institutions must possess a certain level of organizational maturity across multiple domains. The Panamerican Health Organization’s Maturity Model for Information Systems for Health (MM IS4H) provides a comprehensive framework for assessing an organization’s maturity in key areas, including data management, IT infrastructure, governance, knowledge management, and innovation [65]. This model is valuable for evaluating an institution’s readiness to develop and deploy NLP-based systems. Specifically, the MM IS4H maturity levels directly impact an institution’s ability to develop and implement NLP models. At levels 1 and 2, limitations in IT infrastructure, data standardization, and human resources hinder the development of NLP models. In contrast, institutions at level 3 have basic data analysis capabilities and access to core data sets, enabling the training of basic NLP models that can extract insights from structured sources. In contrast, institutions at levels 4 and 5 have formal data governance mechanisms, adequate human resources with the necessary skills, and the capacity to train advanced NLP models to extract valuable information from unstructured text sources.

As highlighted in this work, data availability significantly influences the selection of clinical NLP modeling paradigms. However, paradigm selection requires a more comprehensive assessment, encompassing data availability and the institution’s organizational maturity across multiple domains, including human resources, access to computing power, and infrastructure. While our recommendations offer practitioners validated and robust guidance on paradigm selection, we emphasize the importance of considering additional factors that can impact the successful implementation of clinical NLP in real-world settings. These factors may include, but are not limited to, institutional governance, data governance policies, and available resources, such as personnel expertise and infrastructure. By examining these factors alongside data availability and paradigm selection, engineers can ensure a more comprehensive and effective clinical NLP implementation.

## Conclusion

Our study investigated the impact of data availability on the performance of clinical NLP modeling in simulated settings with varying levels of access to task-specific labeled data and unlabeled environment-specific text. We explored different paradigms, including pre-train, fine-tune and predict, fine-tune and predict, as well as prompt and predict with few-shot learning. The results indicate that choosing foundation models, especially those closer to the target domain, impacts model performance. The Spanish biomedical and clinical RoBERTa model, tailored to the clinical domain, outperformed other models in our experiments. While continuing pre-training with environment-specific data improved model performance, the gains were marginal compared to the computational resources required. The fine-tuning paradigm without additional pre-training proved practical, particularly in settings with limited access to unlabeled data.

In-context learning, using the prompt and predict paradigm, demonstrated its viability in scenarios where there is no labeled data available. The creation of few-shot examples significantly improved performance, highlighting the potential of this approach in data-scarce environments. Our study also revealed a saturation point in performance concerning the amount of training data available. In some instances, minimal data access can still lead to relatively high performance, particularly for less complex tasks. The choice of foundation models, the utilization of available data, and the selection of appropriate modeling paradigms are crucial considerations in clinical NLP tasks. While pre-training and fine-tuning with domain-specific data remain effective, in-context learning with few-shot examples offers a viable solution in settings where labeled data is unavailable.

### Limitations

Our settings were designed to mimic real-world scenarios, but they may reflect biases from local environments, limiting the generalizability of our findings to broader multilingual or cross-cultural contexts. Additionally, our selection of foundation models may not fully capture the diversity of available approaches. Another limitation is the potential bias in the datasets, such as demographic representation, which could affect model performance across different healthcare settings. Variations in patient populations, may influence outcomes, highlighting the need for future work to assess and mitigate dataset biases through fairness-aware training and broader benchmarking.

## Supplementary Information

Supplementary Material 1.

## Data Availability

All datasets used in this publication are available for download. Referral Prioritization: https://huggingface.co/datasets/fvillena/ges, Referral specialty classification: https://huggingface.co/datasets/fvillena/spanish_diagnostics and Named entity recognition: https://huggingface.co/datasets/plncmm/wl.
