# Supplementary material for: NLP modeling recommendations for restricted data availability in clinical settings
Source: BMC Med Inform Decis Mak. 2025 Mar 7;25:116. doi: 10.1186/s12911-025-02948-2 (PMC11889813; doi:10.1186/s12911-025-02948-2)
Supplement: Supplementary file 1 — Supplementary Material 1. [file 12911_2025_2948_MOESM1_ESM.pdf]

1 Prompt templates used for in-context learning

We describe the prompt templates we used to solve each clinical NLP task using in-context learning.

1.1 Referral prioritization

System prompt template En Chile, las garantías explícitas de salud establecen prioridad para un conjunto de problemas de salud. Debes responder en español sólo la palabra "Verdadero" si la enfermedad que te entregue pertenece a uno de los 80 problemas de salud y sólo la palabra "Falso" si la enfermedad no pertenece al conjunto de problemas. Los problemas de salud son: "Accidente Cerebrovascular Isquémico en personas de 15 años y más", "Alivio del dolor y cuidados paliativos por cáncer avanzado ", "Analgesia del Parto", "Artritis Reumatoídea", "Artritis idiopática juvenil", "Asma Bronquial moderada y grave en personas menores de 15 años", "Asma bronquial en personas de 15 años y más", "Cardiopatías congénitas operables en menores de 15 años", "Colecistectomía preventiva del cáncer de vesícula en personas de 35 a 49 años", "Consumo Perjudicial o Dependencia de riesgo bajo a moderado de alcohol y drogas en personas menores de 20 años", "Cáncer Cervicouterino", "Cáncer Colorectal en personas de 15 años y más", "Cáncer Vesical en personas de 15 años y más", "Cáncer de Ovario Epitelial", "Cáncer de mama en personas de 15 años y más", "Cáncer de próstata en personas de 15 años y más", "Cáncer de testículo en personas de 15 años y más", "Cáncer en personas menores de 15 años", "Cáncer gástrico", "Depresión en personas de 15 años y más", "Desprendimiento de retina regmatógeno no traumático", "Diabetes Mellitus Tipo 1", "Diabetes Mellitus Tipo 2", "Displasia broncopulmonar del prematuro", "Displasia luxante de caderas", "Disrafias espinales", "Endoprótesis total de cadera en personas de 65 años y más con artrosis de cadera con limitación funcional severa", "Enfermedad Pulmonar Obstructiva Crónica de Tratamiento Ambulatorio", "Enfermedad Renal Crónica Etapa 4 y 5", "Enfermedad de Parkinson", "Epilepsia no refractaria en personas de 15 años y más", "Epilepsia no refractaria en personas desde 1 año y menores de 15 años", "Esclerosis múltiple remitente recurrente ", "Esquizofrenia", "Estrabismo en personas menores de 9 años", "Fibrosis Quística", "Fisura labiopalatina", "Gran Quemado", "Hemofilia", "Hemorragia Subaracnoidea secundaria a Ruptura de Aneurismas Cerebrales", "Hepatitis C", "Hepatitis crónica por Virus Hepatitis B", "Hipertensión arterial primaria o esencial en personas de 15 años y más", "Hipoacusia Bilateral en personas de 65 años y más que requieren uso de audífono", "Hipoacusia neurosensorial bilateral del prematuro", "Hipotiroidismo en personas de 15 años y más", "Infarto agudo del miocardio", "Infección respiratoria aguda (IRA) de manejo ambulatorio en personas menores de 5 años", "Leucemia en personas de 15 años y más", "Linfomas en personas de 15 años y más", "Lupus Eritematoso Sistémico", "Neumonía adquirida en la comunidad de manejo ambulatorio en personas de 65 años y más", "Osteosarcoma en personas de 15 años y más", "Politraumatizado Grave", "Prevención de Parto Prematuro", "Prevención secundaria enfermedad renal crónica terminal", "Retinopatía del prematuro", "Retinopatía diabética", "Salud Oral Integral del adulto de 60 años", "Salud oral integral de la embarazada", "Salud oral integral para niños y niñas de 6 años", "Síndrome de Dificultad Respiratoria en el recién nacido", "Síndrome de la inmunodeficiencia adquirida VIH/SIDA", "Trastorno Bipolar en personas de 15 años y más", "Trastornos de generación del impulso y conducción en personas de 15 años y más, que requieren Marcapaso", "Tratamiento Médico en personas de 55 años y más con Artrosis de Cadera y/o Rodilla, leve o moderada", "Tratamiento Quirúrgico de Hernia del Núcleo Pulposo Lumbar", "Tratamiento Quirúrgico de lesiones crónicas de la válvula aórtica en personas de 15 años y más", "Tratamiento Quirúrgico de lesiones crónicas de las válvulas mitral y tricúspide en personas de 15

años y más”, ”Tratamiento de Erradicación del Helicobacter Pylori”, ”Tratamiento de Hipoacusia moderada en personas menores de 4 años”, ”Tratamiento de la hiperplasia benigna de la próstata en personas sintomáticas”, ”Tratamiento quirúrgico de cataratas”, ”Tratamiento quirúrgico de escoliosis en personas menores de 25 años”, ”Trauma Ocular Grave”, ”Traumatismo Cráneo Encefálico moderado o grave”, ”Tumores Primarios del Sistema Nervioso Central en personas de 15 años o más”, ”Urgencia Odontológica Ambulatoria”, ”Vicios de refracción en personas de 65 años y más” y ”Órtesis (o ayudas técnicas) para personas de 65 años y más”

User prompt template ¿”¡x¿” pertenece a la lista de 80 problemas de salud priorizados por las garantías explícitas de salud?.

1.2 Referral speciality classification

System prompt template Eres un asistente serio que sólo da respuestas precisas y concisas que recibirá diagnósticos en Español y deberás sólo responder con el nombre de la especialidad en Español a la cual debe enviarse el diagnóstico. Las especialidades disponibles son: TRASTORNOS TEMPOROMANDIBULARES Y DOLOR OROFACIAL, REHABILITACION: PROTESIS FIJA, NUTRICION, GENETICA, ODONTOLOGIA INDIFERENCIADO, CIRUGIA TORAX, CIRUGIA INFANTIL, MEDICINA FAMILIAR, NEUROLOGIA, ONCOLOGIA, OBSTETRICIA, CIRUGIA ADULTO, DERMATOLOGIA, GERIATRIA, OTORRINOLARINGOLOGIA, BRONCOPULMONAR, MEDICINA INTERNA, PERIODONCIA, CARDIOLOGIA, OFTALMOLOGIA, REHABILITACION: PROTESIS REMOVIBLE, ENDOCRINOLOGIA, PEDIATRIA, REUMATOLOGIA, CIRUGIA PLASTICA, ORTODONCIA, CIRUGIA DE MAMAS, CIRUGIA PROCTOLOGICA, GASTROENTEROLOGIA, HEMATOLOGIA, UROLOGIA, ANESTESIOLOGIA, ENFERMEDADES DE TRANSMISION SEXUAL, OPERATORIA, NEONATOLOGIA, NEUROCIRUGIA, CIRUGIA VASCULAR PERIFERICA, GINECOLOGIA, CIRUGIA BUCAL, CIRUGIA MAXILO FACIAL, CIRUGIA ABDOMINAL, CARDIOCIRUGIA, PSIQUIATRIA, INFECTOLOGIA, TRAUMATOLOGIA, ENDODONCIA, MEDICINA FISICA Y REHABILITACION, NEFROLOGIA.

User prompt template ¿A qué especialidad debo enviar el diagnóstico ”¡x¿”?.

1.3 Clinical named entity recognition

System prompt template Eres reconocedor de entidades nombradas que solo debe detectar las entidades en la siguiente lista: ”disease”: ”alteración o desviación del estado fisiológico en una o varias partes del cuerpo, por causas en general conocidas, manifestada por síntomas y signos característicos, y cuya evolución es más o menos previsible”, - medication: ”Medicamentos o drogas empleadas en el tratamiento y o prevención de enfermedades”, - abbreviation: ”Abreviatura”, - body_part: ”Órgano o una parte anatómica de una persona”, - family_member: ”Miembro de la familia”, - laboratory_or_test_result: ”Resultado de laboratorio o test”, - clinical_finding: ”Observaciones, juicios o evaluaciones que se hacen sobre los pacientes”, - diagnostic_procedure: ”Exámenes que permiten determinar la condición del individuo ”, - laboratory_procedure: ”Exámenes que se realizan en diversas muestras de pacientes que permiten diagnosticar enfermedades mediante la detección de biomarcadores y otros parámetros”, - therapeutic_procedure: ”Actividad o tratamiento

que es empleado para prevenir, reparar, eliminar o curar la enfermedad del individuo", Debes responder con el mismo texto de entrada, pero con las entidades nombradas anotadas con etiquetas en la misma línea (¡nombre_entidad¿lorem ipsum¡/nombre_entidad¿), donde cada etiqueta corresponde a un nombre de entidad, por ejemplo: ¡entidad¿Sed ut perspiciatis¡/entidad¿ unde omnis iste natus error sit voluptatem ¡entidad¿accusantium¡/entidad¿. Las únicas etiquetas disponibles son: medication, abbreviation, body_part, family_member, laboratory_or_test_result, clinical_finding, diagnostic_procedure, laboratory_procedure, therapeutic_procedure, no puedes agregar más etiquetas de las incluidas en esa lista. IMPORTANTE: NO DEBES CAMBIAR EL TEXTO DE ENTRADA, SÓLO AGREGAR LAS ETIQUETAS.

2 Extended results

Model	Paradigm	Task	macro-Precision	macro-Recall	Macro-F_1
xlm-roberta	Continue pre-training. fine-tune and predict	Prioritization	89.81 %	88.30 %	89.03 %
xlm-roberta	Fine-tune and predict	Prioritization	89.88 %	87.90 %	88.85 %
xlm-roberta	Continue pre-training. fine-tune and predict	Specialty	77.63 %	52.23 %	52.36 %
xlm-roberta	Fine-tune and predict	Specialty	58.70 %	51.35 %	51.71 %
xlm-roberta	Continue pre-training. fine-tune and predict	NER	11.04 %	19.18 %	13.85 %
xlm-roberta	Fine-tune and predict	NER	9.07 %	14.97 %	11.09 %
roberta-bne	Continue pre-training. fine-tune and predict	Prioritization	89.37 %	88.25 %	88.80 %
roberta-bne	Fine-tune and predict	Prioritization	90.16 %	87.20 %	88.58 %
roberta-bne	Continue pre-training. fine-tune and predict	Specialty	58.28 %	51.02 %	51.65 %
roberta-bne	Fine-tune and predict	Specialty	58.68 %	51.95 %	52.50 %
roberta-bne	Continue pre-training. fine-tune and predict	NER	20.58 %	28.08 %	23.29 %
roberta-bne	Fine-tune and predict	NER	20.67 %	26.41 %	22.59 %
roberta-biomedical-clinical	Continue pre-training. fine-tune and predict	Prioritization	89.17 %	88.54 %	88.85 %
roberta-biomedical-clinical	Fine-tune and predict	Prioritization	90.17 %	87.59 %	88.80 %
roberta-biomedical-clinical	Continue pre-training. fine-tune and predict	Specialty	61.25 %	53.01 %	53.85 %
roberta-biomedical-clinical	Fine-tune and predict	Specialty	61.24 %	53.01 %	53.79 %
roberta-biomedical-clinical	Continue pre-training. fine-tune and predict	NER	33.24 %	43.04 %	37.25 %
roberta-biomedical-clinical	Fine-tune and predict	NER	30.04 %	41.21 %	34.46 %
Llama 2	Prompt and predict (zero-shot)	Prioritization	34.99 %	33.67 %	6.49 %
Llama 2	Prompt and predict (few-shot)	Prioritization	56.42 %	64.11 %	56.70 %
Llama 2	Prompt and predict (zero-shot)	Specialty	25.08 %	37.75 %	31.41 %
Llama 2	Prompt and predict (few-shot)	Specialty	25.19 %	37.85 %	31.91 %
Llama 2	Prompt and predict (zero-shot)	NER	12.46 %	3.82 %	5.31 %
Llama 2	Prompt and predict (few-shot)	NER	26.65 %	11.97 %	15.44 %
Llama 3	Prompt and predict (zero-shot)	Prioritization	51.57 %	67.70 %	36.87 %
Llama 3	Prompt and predict (few-shot)	Prioritization	52.41 %	71.48 %	47.64 %
Llama 3	Prompt and predict (zero-shot)	Specialty	39.65 %	50.54 %	38.49 %
Llama 3	Prompt and predict (few-shot)	Specialty	52.30 %	48.85 %	48.50 %
Llama 3	Prompt and predict (zero-shot)	NER	18.06 %	24.84 %	24.84 %
Llama 3	Prompt and predict (few-shot)	NER	25.27 %	30.50 %	23.15 %

Table 1: Results for each clinical NLP task and each paradigm.
